# An Overview of Scaffolds and Biomaterials for Skin Expansion and Soft Tissue Regeneration: Insights on Zinc and Magnesium as New Potential Key Elements

**DOI:** 10.3390/polym15193854

**Published:** 2023-09-22

**Authors:** Nourhan Hassan, Thomas Krieg, Max Zinser, Kai Schröder, Nadja Kröger

**Affiliations:** 1 Department of Plastic, Reconstructive and Aesthetic Surgery, Faculty of Medicine, University Hospital Cologne, Kerpener Str. 62, 50937 Cologne, Germany; 2 Biotechnology Department, Faculty of Science, Cairo University, Giza 12613, Egypt; 3 Translational Matrix Biology, Medical Faculty, University of Cologne, 50923 Cologne, Germany; 4 Cologne Excellence Cluster on Cellular Stress Responses in Aging-Associated Diseases (CECAD), University of Cologne, 50923 Cologne, Germany; 5 Center for Molecular Medicine (CMMC), University of Cologne, 50923 Cologne, Germany; 6 Department for Oral and Craniomaxillofacial and Plastic Surgery, University of Cologne, Kerpener Strasse 62, 50931 Cologne, Germany

**Keywords:** skin expansion, biomaterials, tissue engineering, metallic alloys, implants

## Abstract

The utilization of materials in medical implants, serving as substitutes for non-functional biological structures, supporting damaged tissues, or reinforcing active organs, holds significant importance in modern healthcare, positively impacting the quality of life for millions of individuals worldwide. However, certain implants may only be required temporarily to aid in the healing process of diseased or injured tissues and tissue expansion. Biodegradable metals, including zinc (Zn), magnesium (Mg), iron, and others, present a new paradigm in the realm of implant materials. Ongoing research focuses on developing optimized materials that meet medical standards, encompassing controllable corrosion rates, sustained mechanical stability, and favorable biocompatibility. Achieving these objectives involves refining alloy compositions and tailoring processing techniques to carefully control microstructures and mechanical properties. Among the materials under investigation, Mg- and Zn-based biodegradable materials and their alloys demonstrate the ability to provide necessary support during tissue regeneration while gradually degrading over time. Furthermore, as essential elements in the human body, Mg and Zn offer additional benefits, including promoting wound healing, facilitating cell growth, and participating in gene generation while interacting with various vital biological functions. This review provides an overview of the physiological function and significance for human health of Mg and Zn and their usage as implants in tissue regeneration using tissue scaffolds. The scaffold qualities, such as biodegradation, mechanical characteristics, and biocompatibility, are also discussed.

## 1. Introduction

The human body is made up of various organ systems that are composed of distinct types of cells within an extracellular matrix (ECM), forming different tissues. These cells are highly complex biochemical, molecular, and electrical reaction chambers that are interconnected within the ECM by cellular receptors and cytoskeletal structures [1]. The human body and its constituent cells are subject to diverse mechanical forces on a daily basis, such as tension, compression, shear, gravity, osmotic pressure, and hydrostatic pressure. The impact of microgravity on cellular biology is also acknowledged, particularly in the context of space exploration [2,3,4].

The skin, being the body’s largest organ, acts as a protective barrier separating the internal and external environment and interacts with a variety of forces and deformations caused by the environment [5]. Cutaneous cell populations must sense mechanical cues and respond appropriately to maintain homeostasis and proper mechanical function [6]. Thereby, ECM plays a crucial role in transmitting applied forces to the cells. These forces trigger mechanosignaling pathways in the cells and elicit various biological responses [7,8,9]. The ECM is produced by fibroblasts, which reside in the dermis and are mainly responsible for the tissue’s mechanical properties. The mechanisms of mechanotransduction are similar among many cell types in the body’s various connective tissues [10]. At the molecular level, these responses may involve changes in the configuration of cell membrane channels or receptor sensitivity, enzymatic and protein synthesis in the cytoplasm, and gene expression in the nucleus. In response to these molecular and biochemical reactions, cells can differentiate, proliferate, migrate, or undergo apoptosis [11]. Mechanical forces are increasingly being leveraged to shape cellular and tissue responses in ways that promote tissue regeneration, scar modulation, and wound healing. In the case of the skin, it possesses the remarkable ability for self-regeneration through the presence of stem cells within the epidermis. However, when faced with deep injuries and severe burns, the natural healing process may prove insufficient, resulting in the development of severe scars, wound contraction, and chronic wounds [12]. Therefore, to overcome extensive mechanical forces, sophisticated surgical techniques have to be used to close the wounds with satisfactory scar formation. These also include the application of skin crafts, skin substitutes, and tissue expansion. In the late 1980s, tissue engineering emerged as a distinct field in response to the pressing surgical challenges that needed to be addressed [13,14]. There are several limitations associated with translational applications in soft tissue engineering strategies, including issues related to cell survival, mechanical challenges such as scaffold collapse and availability, considerations of the microenvironment’s composition, potential induction of malignant behavior, cell migration, and cell exhaustion [15]. Tissue expansion is a widely used surgical technique aimed at growing additional skin to address various reconstructive needs such as birth defects, burn injuries, or cancerous breasts [16]. The technique of tissue expansion has been in practice for over three decades and has proven to be a valuable tool in reconstructive surgery across various anatomical regions [17]. One of the major hurdles encountered by reconstructive surgeons is the scarcity of viable soft tissues for such procedures [18]. As a result, there is a growing interest in tissue-engineered skin substitutes as alternative approaches to traditional wound healing, skin expansion strategies, and tissue regeneration [14,19]. In this regard, we highlight in this review several types of implants used in translational applications in soft tissue engineering strategies, including magnesium and zinc, as remarkable promising materials to be used in the field of soft tissue engineering and skin expansion.

## 2. Mechanotransduction in Skin

**Figure 1:**
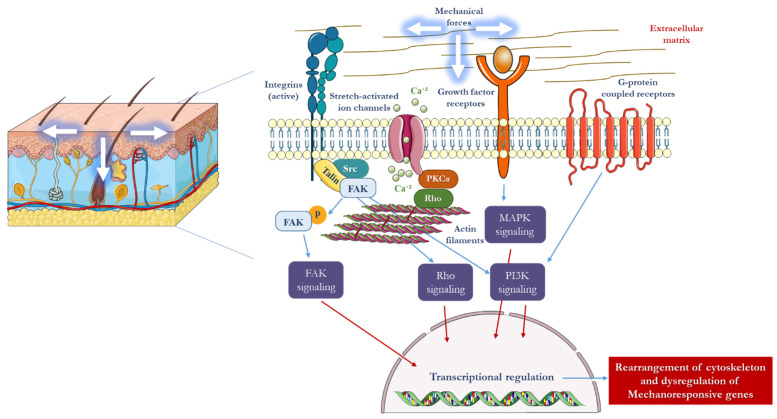
In response to mechanical force, a number of intracellular signaling pathways are initiated in mechanotransduction. Membrane-bound mechanosensory complexes such as stretch-activated ion channels, growth factor receptors, integrins, and G-protein-coupled receptors play a crucial role in sensing mechanical strain. In fibroblasts and keratinocytes, where matrix-integrin activation takes place in focal adhesion complexes, FAK is crucial. The mechanical force that is transferred across the cell membrane activates downstream biochemical pathways, such as calcium-dependent targets, nitric oxide (NO) signaling, MAPKs, Rho GTPases, and phosphoinositol-3-kinase (PI3K). When these signals come together, transcription factors are induced to activate mechanoresponsive genes in the nucleus [29]. Parts of the figure were drawn using elements from Servier Medical Art. (https://creativecommons.org/licenses/by/3.0/) (accessed on 16 May 2022).

Mechanically sensitive cells, especially fibroblasts, have three types of mechanical sensors at the cell membrane: integrins, G protein-coupled receptors, and stretch-activated ion channels. Furthermore, the cytoskeleton, which provides overall structural support to the cell, can sense deformations through conformational changes, resulting in an additional sensing mechanism [20]. Activation of mechanical sensors directly triggers intracellular signaling pathways, which often activate secondary messengers, such as growth factors [21]. Growth factor receptors located at the cell membrane represent another important sensing mechanism. In response to mechanical stimuli, various cytokines are expressed in connective tissues, including transforming growth factors (TGF-beta), interleukins, fibroblast growth factors (FGF), vascular endothelial growth factors (VEGF), platelet-derived growth factors (PDGF), and tumor necrosis growth factors (TNF-alpha) (Figure 1). These cytokines are particularly important in connective tissues and contribute to various physiological processes [10].

The maintenance of the ECM by dermal fibroblasts involves a constant cycle of collagen and proteoglycan deposition and degradation of the collagen network through matrix metalloproteinases (MMP). Among the various mechanical cues that fibroblasts experience, tension is the most physiologically relevant. Thus, in vitro studies have primarily focused on examining the response of fibroblasts to uniaxial and biaxial strain loading conditions using flexible two-dimensional constructs [22]. In order to better simulate the in vivo environment, mechanical strain has also been applied to fibroblasts embedded within three-dimensional collagen gels [23]. The application of tensile strain to the ECM induces conformational changes in the cytoplasmic tails of the main ECM receptors and the integrins, which activate kinases such as focal adhesion kinase (FAK). FAK activation is then linked to mitogen-activated protein kinase (MAPK) pathways inside the cell [24]. The downstream effects of FAK activation include pro-inflammatory signaling, collagen production, and reduced apoptosis (Figure 1) [25]. Aside from direct mechanical sensing, additional signaling pathways, particularly TGF-beta, play a critical role in controlling how the ECM is remodeled by fibroblasts [26]. TGF-beta exposure results in the up-regulation of collagen genes and the downregulation of the Bax apoptotic gene in fibroblasts [23]. Other mechanical stimuli, including the microstructure and composition of the ECM, also influence fibroblast behavior. For instance, fibroblasts have been found to migrate preferentially along fiber directions, demonstrating the importance of ECM factors in regulating fibroblast behavior [27,28].

While the dermis is commonly considered the main load-bearing layer of the skin, it is important to note that keratinocytes in the epidermis also demonstrate mechanosensitivity. Deformations of the dermis are conveyed to the epidermis through hemidesmosome junctions at the basement membrane. Forces are then transmitted to the cytoskeleton inside the keratinocytes via adherens junctions between neighboring cells. Subsequently, intracellular signaling is induced by deformations of the overall cell shape that affect keratinocyte mitosis [30]. Stretch-activated ion channels represent another significant type of mechanoreceptor in the epidermis [31]. When activated downstream of a mechanical stimulus, growth factor receptors in keratinocytes, such as epidermal growth factor (EGF), play a crucial role in controlling cellular proliferation [6]. Experiments on cultured keratinocytes have demonstrated increased mitosis in response to strain [8]. More recently, in vitro studies have explored engineered skin, where both the dermal and epidermal layers respond to strain in a coordinated mechanobiological manner [32].

**Figure 2:**
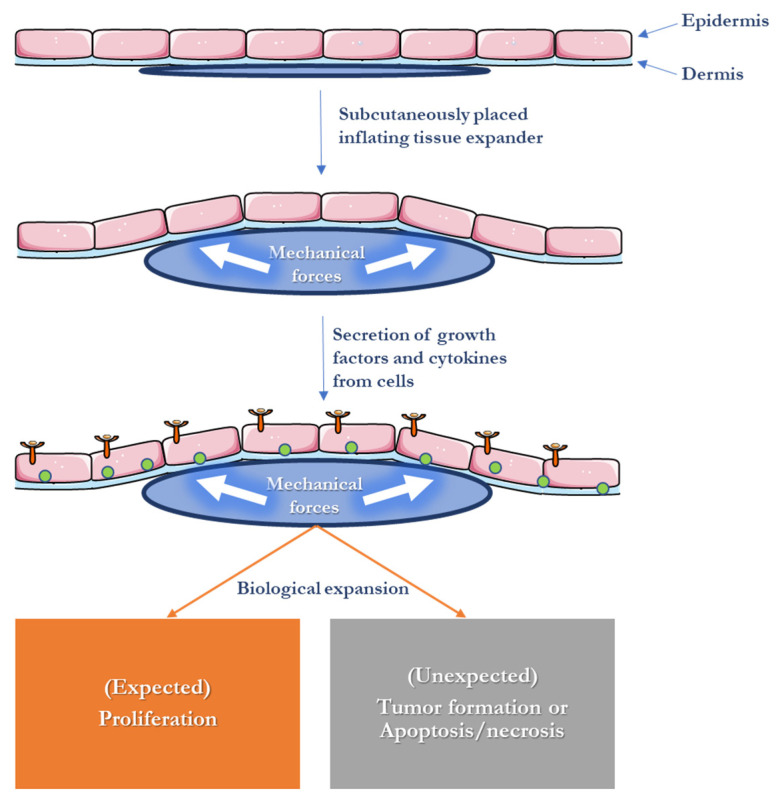
Schematic sequence of tissue expander inflation. Initially, the skin is in a state of rest, with no tension present (**top**). A tissue expander which is inserted underneath the skin, starts to inflate between the epidermis and dermis layers and the hypodermis. Upon inflation of the expander, the skin becomes taut and stretched (**middle**). The mechanical stretching results in cellular proliferation, leading to either skin growth and proliferation or apoptosis or tumor formation. Eventually, the skin grows enough to return to its original state of rest, with no tension (**bottom**) [34]. Parts of the figure were drawn using elements from Servier Medical Art. (https://creativecommons.org/licenses/by/3.0/) (accessed on 16 May 2022).

Mechanotransduction in vivo has mostly been investigated in relation to tissue expansion, which involves implanting a subcutaneous balloon that is persistently inflated over many weeks in order to grow skin [17,33]. By stretching the skin beyond its normal capacity, the expansion process leads to tissue growth (Figure 2) [34]. The ultimate aim of tissue expansion is to avoid donor site morbidity, using the newly created skin as a vascularized flap to reconstruct soft tissue defects during a second operation [35]. However, the procedure does have some limitations, such as potential failures and complications, as well as the need for training and skill development to effectively plan and execute tissue expansion [36]. The technique is also not appropriate for pre-existing open wounds, which represents its biggest drawback [37]. The analysis of expanded tissues has shown an increase in keratinocyte proliferation, activation of the MAPK pathway, and an increase in collagen deposition [38,39,40]. Interestingly, the grown tissue has similar properties to native, unexpanded skin.

## 3. The Influence of Mechanical Forces on the Structure and Function of the Skin

Mechanotransduction is the process of converting physical forces into biochemical signals that trigger cellular responses. The mechano-responsiveness of cellular complexes, such as TGFβ/Smad, integrin, and calcium ion pathways, has been demonstrated (Figure 1) [41]. These signals are transmitted into the cell, ultimately reaching the nucleus. In vitro models have shown that mechanical strain can upregulate matrix remodeling genes and downregulate normal cellular apoptosis through an Akt-dependent mechanism, leading to increased production of extracellular matrix [42,43]. The skin is exposed to stretching forces both under normal physiological conditions, such as pregnancy, and through external means, such as tissue expansion using soft tissue expanders, external skin stretching devices, and distraction osteogenesis using external devices in hard tissue.

Skin stretching devices and techniques are useful for treating open wounds in surgery and are referred to as external tissue expanders [44]. Typically, they use hooks, sutures, wires, or loops to engage the skin and apply a mechanical force of tension to promote the approximation of wound edges through mechanical creep over time. The stretched skin edges then heal spontaneously during a consolidation period while the stretching device remains engaged. In most cases, the mechanically stretched skin is surgically freshened at the edges and closed after the device is removed. The impact of mechanical skin stretching devices or techniques on healing wounds is increasingly gaining attention [45].

The process of wound healing engages different types of cells, including inflammatory cells, keratinocytes, fibroblasts, myofibroblasts, and endothelial cells. These cells play a pivotal role in cutaneous healing, and they react to mechanical forces by initiating a cascade of events and pathways at both the cellular and molecular levels. This process takes place in the context of the tensegrity model, which involves the cytoskeletal framework being anchored in ECM [39,46]. The structurally interconnected cells respond to mechanical stimuli. Mechanotherapies are wound healing treatments that use mechanical forces to enhance the healing process. Examples of these therapies include micro-deformational wound therapy (MWT), such as negative pressure wound therapy (NPWT) [47,48], shock wave therapy [49,50], ultrasound [51], and electrotherapy [52]. Each therapy uses a different form of mechanical force to stimulate the cells and tissues involved in wound healing. The effects of NPWT have been extensively studied [53]. When suction is applied, the sponge collapses, causing the wound to shrink. This results in macro- and micro-deformation at the wound-sponge interface, which triggers mechanosignaling in a closed wound-healing environment [54]. This therapy has various biological effects, such as increased gene expression of leucocyte chemoattractants, proliferation and migration of epithelial cells and dermal fibroblasts, decreased activity of matrix metalloproteinases, and increased micro-vessel density [55,56,57,58]. As a result, NPWT promotes moist wound healing, angiogenesis, collagen synthesis, and the breakdown of dead tissue and fibrin [59]. Currently, NPWT is extensively utilized to expedite the healing of different types of wounds across various anatomical locations.

Scar formation is a significant concern after skin injury both functionally and aesthetically [60]. In contrast to the scarring process, which occurs in extra-uterine life, fetal wound healing follows a regenerative process [61,62,63]. This has sparked considerable research interest in the conversion from regenerative healing to scarring, with the ultimate goal of achieving scarless wound healing. Currently, there is growing interest in mechanobiology and scar research, with the aim of utilizing mechanotherapy to prevent and treat abnormal scarring [64]. The relationship between tension and scar growth has been observed clinically, particularly in keloid formation, where different shapes and configurations are seen, often associated with tension [65,66,67,68]. Animal models have demonstrated that mechanosignaling plays a role in the fibroproliferative response to tension [64]. Mechanotherapy, which aims to alter stimuli, processing, or reception, offers strategies to deal with these tension forces, such as skin stabilization by paper and silicone tape, multilayered suturing and plication, flaps, z-plasty, and the addition of radiotherapy [69]. The goal is to reduce skin tension, which is the source of cyclically applied mechanical forces in daily locomotion, and ultimately decrease skin inflammation.

Skin tissue engineering encompasses a multidisciplinary approach, incorporating diverse fields such as biochemistry, polymer chemistry, and stem cell research. The objective of skin tissue engineering is to synergize the expertise from these disciplines in order to develop a substitute that can be efficiently produced and effectively restore the skin’s natural functional, mechanical, and aesthetic characteristics. This involves regenerating ECM to provide support and guidance, improving graft take by establishing a vascular network, and restoring skin appendages for functions such as thermoregulation and sensitivity, as well as the various cell types required for protection. The objective can be achieved through two primary methods. The first method involves creating a biodegradable scaffold that is sophisticated enough to release a specific set of signaling molecules in a controlled manner, which can facilitate the migration, adhesion, and, ultimately, regeneration of skin cells. The second method involves designing a basic, temporary scaffold that can serve as a carrier for stem cells or undifferentiated cells to encourage skin regeneration [70]. Both methods necessitate the creation of a 3D environment that can support cell interactions and foster wound healing.

Even though we have emphasized how important mechanical forces and load-induced events are in skin tissue engineering, it is still critical to provide a quantitative perspective, especially when considering various tissues [71]. Quantitative data can provide a more thorough understanding of how different tissues are impacted by mechanical forces, resulting in more accurate and successful tissue engineering techniques [72]. Depending on the tissue under examination, there are several ways to quantitatively present mechanical forces, including tensile strength and strain, strain distribution, shear forces, compression and stress relaxation, fluid flow and perfusion, frequency and magnitude of mechanical loading, microenvironmental stiffness, and biomechanical properties of biomaterials [73,74,75,76]. Thus, researchers are exploring numerous scaffold materials and techniques to meet the requirements of skin tissue engineering.

## 4. Skin Expansion in Reconstructive Surgery

Soft-tissue expanders have emerged as a pre-augmentation technique in implant surgery to circumvent the complications associated with bone grafting procedures [77,78]. The principle of soft tissue expansion is rooted in the biological response of various soft tissues, including skin and mucous membranes, to mechanical forces by producing true tissue growth (cell proliferation) [17]. This phenomenon is evident in various situations, including pregnancy, muscle growth, obesity, and specific cultural practices such as lip and neck expansion in African customs [79]. Tissue expansion offers a remarkable approach to cultivating skin that closely resembles the neighboring healthy skin in terms of texture, color, and hair-bearing characteristics, thereby minimizing scarring and the potential for rejection [80]. The technique of soft tissue expansion is clinically useful in several ways, including preoperative expansion of oral mucosa for large bone augmentations, as well as the intra-oral repair of clefts in the lip and/or palate. Its applications have been popular in plastic surgery since 1976 [81]. Moreover, they are well-established for a variety of indications, such as correcting skin burns after burn wounds, scars, alopecia, congenital nevi, and post-mastectomy breast reconstruction [82,83,84,85]. It has also evolved into one of the principal surgical procedures for creating skin flaps to resurface large congenital defects of the skin, such as giant nevi and vascular anomalies [86,87]. In recent years, this concept has also been introduced in orthopedics, where it was successfully used in a clinical report to achieve skin closure in open fractures using an “external” soft tissue expander. The expansion of soft tissues can reduce the need for periosteal incisions and promote passive flap closure while generating tissues with appropriate color match and texture similar to the original tissues [88].

In 1957, Neumann developed soft tissue expanders using a subcutaneous rubber balloon to repair an ear defect. However, it was not until the early 1980s that soft tissue expanders regained significant interest, particularly in breast reconstruction [89] and the treatment of burns [90]. Early expanders consisted of silicone rubber and featured an external valve that allowed for manual inflation through sequential injections. The extent of soft tissue expansion achieved with conventional expanders has been documented to be influenced by factors such as the specific tissue being expanded and the configuration of the expanders themselves [91,92]. Studies have shown that tissue gain is more pronounced with rectangular and crescent-shaped expanders than with round-based ones [79]. Although conventional soft tissue expanders have shown positive results, they have several disadvantages. The intermittent inflations required for conventional expanders can increase the treatment time by several months and cause pressure peaks, leading to a decrease in tissue vascularity [93] and a higher risk of expander perforation through the soft tissues [88]. The reduction in local oxygen partial pressure increases the risk of expansion failures, and serial injections can result in increased treatment costs, morbidity, and risks for adverse effects [82]. Despite these drawbacks, conventional expanders are still utilized in plastic surgical procedures. However, their use is limited in craniofacial defects due to the aforementioned shortcomings [94].

A self-inflating osmotic soft tissue expander was developed by Austad & Rose (1982) to overcome the drawbacks of conventional soft tissue expanders. It was designed without an external port, and repetitive inflations were not necessary [95]. The new expander was made of a semi-permeable silicone membrane that contained a hypertonic sodium chloride solution. The expansion of the expander and subsequent growth of soft tissue were facilitated by a continuous influx of body fluids driven by an osmotic gradient. This led to an increase in the volume of the expander and the growth of surrounding soft tissues. However, the device had several drawbacks, such as leaks from the expander shell to the surrounding tissues, causing tissue necrosis. Wiese introduced a unique self-inflating osmotic soft tissue expander composed of hydrogel, comprising a polymer network and a variable aqueous component [96,97,98]. This expander, known as Osmed^®^ (Ilmenau, Germany), was developed in 1999 and became the first commercially accessible self-inflatable osmotic expander. It received FDA approval in 2001 and has been available in the market since then. Osmotic expanders eliminate the need for repeated injections and instead inflate continuously by osmotic gradients without requiring any additional interventions. This consistent expansion, in contrast to intermittent inflation, stimulates the generation of new cells, tissue growth [92], and a greater final tissue gain [81,88,99].

The biomaterials used in the hydrogel expanders are the same as those used in contact lenses, providing high biocompatibility and causing no adverse effects such as toxicity, immune reactions, infections, or systemic manifestations [98]. Moreover, they do not provoke any inflammatory responses in the surrounding soft tissues, which is a crucial feature. The presence of methacrylate in ionic hydrogels enhances their osmotic potential, leading to a greater swelling capacity compared to non-ionic hydrogels [96,97,98]. The incorporation of “methyl” methacrylate, specifically in the osmotic hydrogel expanders, results in a higher swelling ratio when compared to “hydroxyethyl” methacrylate [100].

The polymer network of the hydrogel expander is insoluble in water due to the presence of cross-links, making it able to retain large volumes produced by swelling without dissolving [101]. Varga et al. (2009) sought to investigate alternative biomaterials and introduced a hydrogel osmotic soft tissue expander composed of acrylic acid (AAc), acrylamide (AAm), or N-isopropylacrylamide (NIPAAm) [102]. Among these, NIPAAm hydrogels were found to be the most suitable for plastic and reconstructive surgeries in terms of their biological and mechanical properties, although they have only been tested in vivo and require further validation in clinical trials. The next section will delve into the techniques used in scaffold fabrication employed in tissue engineering, shedding light on the advancements, challenges, and future prospects in this exciting field.

## 5. Scaffold Fabrication Methods Used for Tissue Engineering

Numerous techniques have been devised for constructing and fabricating scaffolds in tissue engineering. The choice of technique depends on the specific properties of the materials employed and the desired characteristics of the scaffold. These methods can be classified into conventional and advanced techniques [103].

Conventional techniques encompass several methods, including solvent-casting and particulate-leaching techniques, which entail the utilization of a polymer solution blended with salt particles of precise dimensions. Subsequent to solvent evaporation and immersion in water, the salt particles dissolve, creating a porous structure [104]. However, gas foaming involves subjecting molded biodegradable polymers to high pressures with gas-foaming agents such as CO_2_, nitrogen, water, or fluoroform. The polymers become saturated, leading to the nucleation and expansion of gas bubbles within the polymer matrix, typically ranging in size from 100 to 500 μm [105,106]. Moreover, phase separation entails the rapid cooling of a polymer solution, leading to its separation into two separate phases: a polymer-rich phase and a polymer-poor phase. The polymer-rich phase solidifies, while the polymer-poor phase is removed, resulting in the creation of a porous polymer network with high permeability [107]. In melt molding, a combination of polymer powder and porogen components is introduced into a mold, which is then subjected to elevated temperatures beyond the glass-transition temperature of the polymer, accompanied by the application of pressure. This process causes the raw materials to fuse together, forming a scaffold with a predetermined external shape. After removing the mold, the porogen is washed away, leaving behind a porous scaffold that is subsequently dried [108]. Freeze drying, also known as lyophilization, offers a method for the production of polymeric porous scaffolds. The process involves two stages. Initially, the polymer solution is cooled to a specific temperature, causing all components to freeze. During this freezing stage, ice crystals form from the solvent, prompting the polymer molecules to aggregate within the interstitial spaces. In the subsequent phase, the solvent is eliminated by applying a pressure lower than the equilibrium vapor pressure of the frozen solvent. As the solvent undergoes sublimation, a dry polymer scaffold with a well-connected porous microstructure is left behind. The porosity of the scaffolds is contingent upon the concentration of the polymer solution, while the freezing temperatures affect the distribution of pore sizes. In addition to its use in fabricating porous scaffolds, this technique finds application in drying biological samples to safeguard their bioactivities [109,110].

On the other hand, electrospinning and 3D printing technologies are considered advanced techniques in scaffold fabrication. The former methodology is a fabrication technique that utilizes electrical charges to create ultrafine fibers on a nanometer scale. It has found extensive application in the production of porous scaffolds with nanofibrous structures, closely resembling the architecture and biological properties of the native extracellular matrix [111]. This versatile method enables the generation of fibers ranging from 2 nm to several micrometers in diameter, utilizing solutions composed of both natural and synthetic polymers. The resulting scaffolds exhibit small pore sizes and possess a high surface area-to-volume ratio, making them suitable for various biomedical applications [103,112]. Furthermore, 3D printing technologies encompass a range of methods employed for scaffold fabrication, utilizing CAD/CAM technology (computer-aided design/computer-aided manufacturing) [113]. These techniques serve as viable alternatives to address the drawbacks associated with conventional approaches, such as the utilization of cytotoxic solvents and limited control over porosity. By leveraging this technology, it becomes possible to create patient-specific scaffolds with precise shapes guided by computed tomography (CT) images [114]. Multiple 3D printing technologies exist, each distinguished by their unique construction methods and materials employed during the production process [115]. To achieve successful outcomes in the fabrication of skin substitutes, understanding the properties and characteristics of these materials is crucial, which will be highlighted in the next section.

## 6. Materials Used for Skin Tissue Engineering

Biomaterials are substances that have been specifically designed to assume a particular form, either independently or as part of a more complex system, to influence and guide therapeutic or diagnostic procedures in the field of human or veterinary medicine by controlling their interactions with living systems [49]. Recently, due to the increasing aging of the world’s population, there has been a rise in bone-related diseases and fractures, necessitating treatments that include implants with or without complementary functionality, such as biocompatibility, biodegradability, and antibacterial activity for infection control or growth hormones. To ensure an implant’s success, longevity, and desired function, it is essential to select a suitable biomaterial for the proposed application. These can be classified into natural and synthetic biopolymers, bimetals, bioceramics, and biocomposites.

### 6.1. Natural Materials

Natural materials, including silk, collagen, elastin, chitosan, and fibronectin, have garnered considerable interest in the development of skin substitutes. The utilization of these biologically-derived components offers a significant advantage, as they enable the creation of scaffolds that are both biocompatible and biodegradable. Moreover, the degradation products resulting from the breakdown of these natural polymers are non-toxic, further enhancing their suitability for biomedical applications [70]. Additionally, natural polymers contain peptides that have evolved over time to provide signals that promote wound healing. However, natural polymers have certain drawbacks, such as batch-to-batch variation, the potential for immune rejection, and the risk of pathogen transfer.

#### 6.1.1. Silk

Despite being utilized as sutures in clinical practice for centuries, silk has only recently garnered significant attention as a natural biomaterial for tissue engineering. Silk exhibits unique properties as a lightweight polymer, possessing a tensile strength comparable to that of Kevlar 49, which is an aramid fiber used in composite materials with polymeric organic materials as reinforcement. Notably, silk is also highly elastic and requires a greater amount of energy to break compared to Kevlar 49 [116,117]. Moreover, silks exhibit thermal stability of up to approximately 250 °C, enabling processing at a broad range of temperatures [118]. Silk fibers commonly studied in the field include cocoon silk derived from the silkworm Bombyx mori and dragline silk obtained from the spider Nephila clavipes [119,120,121,122]. The process of silk fiber formation involves the coating of a filament core protein known as silk fibroin with a sericin protein-based adhesive substance [116]. Structurally, both B. mori and N. clavipes silks exhibit distinct blocks of hydrophobic and hydrophilic amino acid sequences [123,124]. The hydrophobic blocks form β-sheets or crystals through hydrophobic interactions or hydrogen bonding, which provide the silk fibroin’s tensile strength, while the less ordered hydrophilic blocks contribute to its elasticity and toughness [119,120,121]. The hydrophobic segments within silk fibroin-like proteins are utilized in genetic engineering approaches to modify host systems, including yeast, E. coli, plant, and mammalian cells. This genetic manipulation leads to the production of recombinant proteins that resemble silk fibroin and exhibit low water solubility, primarily attributed to their inherent hydrophobic nature [122,125,126]. Numerous studies have demonstrated that silkworm silk can foster the attachment and growth of human fibroblasts [127,128,129,130]. Nevertheless, sericin has been identified as a primary cause of adverse immune reactions [131]. The successful elimination of sericin and subsequent regeneration of silk fibroin have resulted in the development of biocompatible [131,132,133], hemocompatible [134], and materials that possess excellent oxygen and water permeability [135]. Furthermore, the utilization of silk fibroin films and composites in wound dressings has demonstrated enhanced healing capabilities in vivo [136,137].

#### 6.1.2. Chitosan

Chitosan, a linear polysaccharide made up of glucosamine and N-acetyl glucosamine units linked by β (1–4) glycosidic bonds, is produced by the partial deacetylation of chitin, the second most abundant natural polymer found in the exoskeletons of crustaceans [138]. Its hydroxyl and amino groups can be modified to synthesize different derivatives of chitosan [139,140,141]. The extent of deacetylation, which can vary from 30% to 95% depending on the source and preparation method, also influences the molecular weight and the quantity of glucosamine present [142,143]. While chitosan is insoluble in aqueous solutions with pH above 7, its protonated free amino groups on glucosamine make it soluble in dilute acids (pH < 6.0) [144]. However, the presence of cationic groups on chitosan poses difficulties for techniques such as electrospinning, as it necessitates a solvent capable of forming a salt with chitosan to disrupt the interactions between adjacent chitosan molecules [145,146,147,148]. These cationic groups also enable pH-dependent electrostatic interactions with anionic glycosaminoglycans (GAG) and proteoglycans [149]. Despite its challenges, chitosan has been shown to support the attachment and growth of cells, making it a promising material for tissue engineering. Chitosan has garnered interest in skin tissue engineering due to its biocompatibility, biodegradability, and bioactivity [150,151]. Furthermore, its capacity to enhance hemostasis, expedite tissue regeneration, and stimulate collagen synthesis by fibroblasts has established it as a valuable polymer [152,153,154,155]. Furthermore, the properties of chitosan are not lost when used to create double-polymer scaffolds. For example, when chitosan and alginate were combined to form a polyelectrolyte complex membrane, it demonstrated enhanced stability against pH changes compared to each material alone, leading to the development of more efficient controlled-release membranes [156]. This capability to retain properties after blending is beneficial in skin tissue engineering because it enables the combination of various materials to generate a scaffold with improved capabilities.

#### 6.1.3. Collagen Type I

Collagen refers to a group of proteins that have a characteristic triple helix structure consisting of three polypeptide chains. These proteins can be classified based on their structure and organization into various types such as fibril-forming, fibril-associated, network-forming, anchoring fibril, transmembrane, basement membrane, and others, each with unique functions. Collagens are known to provide functional properties that promote cell attachment and proliferation [157]. Collagens exhibit a distinct structural pattern consisting of a right-handed triple helix, where each alpha chain forms an elongated left-handed helix with a pitch of 18 amino acids per turn [158]. Collagen molecules can exist as homotrimers or heterotrimers, with the chains staggered by one residue compared to one another and coiled around a central axis [159,160]. The presence of glycine at every third residue facilitates close packing of the alpha chains around this axis, with the bulkier side chains of other amino acids located in the outer positions [161,162]. In tissues such as skin, bone, and articular cartilage, a dynamic 3D environment is created by networks of fibril-forming collagens, including collagen types I, II, IV, V, and XI [161]. These collagens are capable of assembling into highly oriented supramolecular aggregates and display a banding pattern with a periodicity of around 70 nm when observed under SEM [163].

#### 6.1.4. Elastin

The field of skin tissue engineering has shown a growing interest in elastin, a natural polymer. The monomer form, tropoelastin, cross-links to produce the insoluble biopolymer elastin [164]. Tropoelastin is encoded by a single-copy gene located in the 7q12.2 region in humans [165] and is characterized by alternating hydrophobic and hydrophilic domains [166]. These domains have different functions, with the hydrophobic domains responsible for monomer association and elastic function, while the hydrophilic domains facilitate polymerization through cross-linking [167]. The presence of elastin is commonly observed in elastic tissues such as skin, lungs, large arteries, and tendons [168]. Elastin is synthesized by a variety of cell types, including endothelial cells, fibroblasts, and smooth muscle cells [169]. Elastogenesis mainly occurs during the late fetal and early neonatal stages, and there is a limited turnover of elastin in healthy adult tissues. The durability of elastin is noteworthy as it has a half-life of around 70 years [170]. Elastic fibers in the body comprise two primary components, with elastin forming the core, encased within a sheath of microfibrils measuring approximately 10–12 nm in width [171]. Elastin is renowned for conferring flexibility, elasticity, and durability to the skin while simultaneously controlling its texture and quality [170]. When skin is damaged, elastin levels have been found to decrease or be absent, resulting in the reduced suppleness of scar tissue [172].

Elastin exhibits structural properties and inherent cell signaling properties such as chemotaxis, cell attachment, proliferation, and differentiation [166,173]. Due to these unique characteristics, elastin is an attractive polymer for skin tissue engineering, as it may improve the elasticity and cell-scaffold interactions of a skin substitute. Additionally, the presence of enzymatic elastin products has been found to stimulate elastin and collagen production. For example, cultures of dermal fibroblasts with elastin products showed a significant increase in elastin and collagen fiber production, as well as an increase in elastic fiber deposition in skin explants. This effect was also observed in human dermal fibroblasts (HDFs) injected into athymic nude mice, resulting in increased elastic fiber production [174].

#### 6.1.5. Silicon

Silicon plays a crucial function as a biomaterial in supporting tissue regeneration. It is a remarkable component for consideration in the field of tissue engineering due to its distinct qualities and interactions with biological systems [175,176]. Silicon is extremely biocompatible in a variety of forms, including silicon-based ceramics and nanoparticles. This indicates that it can be utilized in close contact with biological tissues without having negative effects [177]. When considering its application as an implant or scaffold material in tissue engineering, its biocompatibility is especially beneficial. Moreover, Silicon has been reported to increase collagen formation, which is an important component of the extracellular matrix (ECM) [178]. Since the ECM offers structural support and signals for cell development and differentiation, this characteristic is crucial for tissue engineering, and its results may be improved by increased collagen production [179]. Silicon has been incorporated into a variety of biomaterials, including hydroxyapatite and bioglass, according to previous studies [180,181]. A favorable microenvironment for tissue regeneration is created by silicon-doped biomaterials, which offer a controlled and continuous release of silicon ions [182,183]. This controlled release mechanism of silicon ions ensures that the beneficial effects are delivered over an extended period, aiding in tissue healing. According to recent studies, silicon-based materials may have natural antibacterial characteristics [184]. For successful regeneration in tissue engineering, infection control is crucial. The possible antibacterial properties of silicon could be a useful addition to biomaterials, lowering the danger of post-implantation infections [184].

### 6.2. Synthetic Bioresorbable Polymers

Synthetic polymers, which are manufactured and easily obtainable, have gained attention in skin tissue engineering. Biodegradable, biocompatible, and bioresorbable synthetic polymers are preferred as they can be naturally degraded and eliminated without surgical intervention. Their predictable mechanical properties, such as tensile strength, offer an advantage in producing reliable treatment outcomes. Nevertheless, synthetic polymers do not possess the inherent biological signals present in natural polymers. Numerous synthetic polymers, including poly(lactic acid) (PLA), poly(glycolic acid) (PGA), and polycaprolactone (PCL), are being researched for use in skin tissue engineering [185].

#### 6.2.1. Polycaprolactone (PCL)

Polycaprolactone (PCL) is an aliphatic polyester that undergoes biodegradation through hydrolysis and has obtained approval from the Food and Drug Administration [186,187]. Despite being synthesized in the 1930s, it has recently regained attention in the field of tissue engineering due to its biocompatibility, high tensile strength, and controllable biodegradability [188]. The degradation rate of PCL can be adjusted and can range from several months to years based on factors such as molecular weight, degree of crystallinity, and degradation conditions of the polymer [189,190]. Furthermore, the degradation products of PCL are non-toxic, in contrast to other synthetic polymers, such as PLA, which may induce mild inflammation [163]. Clinical trials have been conducted on subcutaneously implanted PCL capsules, showing that PCL was well-tolerated for over 40 weeks, and other studies have used PCL as a drug release vehicle [191]. In one study, an ultrathin PCL film was developed as a wound dressing and tested in rat and pig models. The results showed that the PCL films had a lower level of fibrosis compared to non-dressed wounds. The PCL films did not induce inflammation, and the wound dressing supported normal wound healing in both partial and full-thickness wounds [187].

#### 6.2.2. Poly(d,l-lactic-co-glycolic acid) (PLGA)

PLGA is a group of biodegradable and biocompatible polymers approved by the FDA, which has gained popularity due to its extended clinical use and ability to provide sustained drug delivery despite its mild inflammatory degradation products. The copolymer is made up of equal parts of PGA and PLA, which contain both the optically active d and l enantiomers of PLA, with PDLA and PLLA being asymmetrical α-carbons, respectively. PLLA can be highly crystalline, PDLA can be completely amorphous, and PGA is highly crystalline. The solubility of PLGA is widespread, encompassing commonly used solvents such as acetone and ethyl acetate [192,193]. Under aqueous conditions, PLGA degrades through hydrolysis at its ester linkages. Consequently, by including more of the less hydrophilic PLA, water absorption can be reduced, leading to slower degradation rates [194]. Moreover, factors such as molecular weight and storage temperature have been demonstrated to influence the physical properties of PLGA, including mechanical strength [195].

### 6.3. Absorbable Metallic Materials

**Figure 3:**
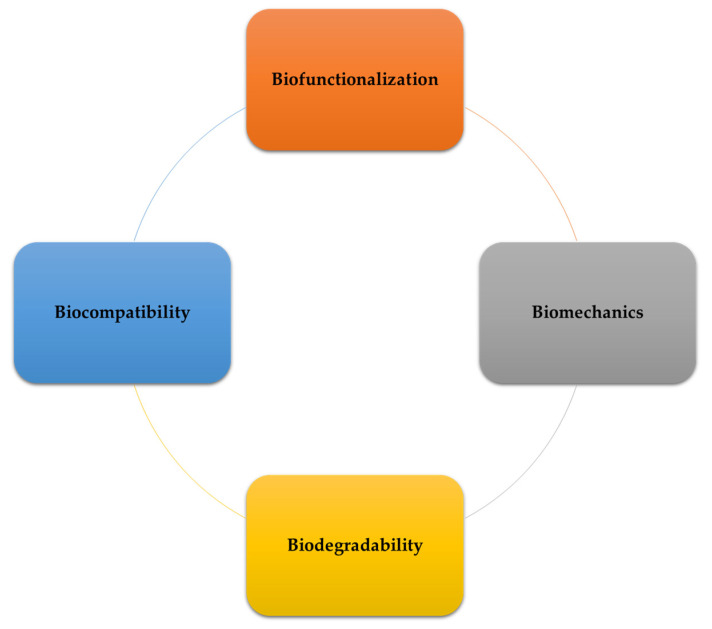
Essential characteristics of an optimal metallic implant [207].

It is possible to significantly enhance the effectiveness of tissue regeneration by introducing certain physiologically active substances, such as metal elements, growth factors, peptides, genes, and stem cells [196,197]. Metal elements play important roles in the structure or expression of several biomacromolecules, including proteins and enzymes, as vital parts of the human body [198,199,200]. Metals are highly desirable for load-bearing implants due to their excellent mechanical properties and biocompatibility. Numerous studies have demonstrated that the regulation of various metal elements, which are essential for cytokine regulation and immunological processes, is closely associated with the tissue regeneration process [201,202,203]. Furthermore, certain metal particles possess inherent antibacterial properties that can effectively combat invading pathogens [204,205]. As a result of advancing research into the mechanisms underlying metal elements in soft tissue regeneration, wound repair techniques incorporating metal elements have gained significant attention [206]. Ideal biomaterials must encompass considerations of biocompatibility, biomechanics, biodegradability, and biofunctionalization (Figure 3) [207]. Currently, stainless steels, titanium, and cobalt-chromium-based alloys are the most commonly used metallic biomaterials [208], and strontium (Sr), iron (Fe), zinc (Zn), and magnesium (Mg) are the most commonly utilized biodegradable metals in clinical practice [209]. Titanium alloys have gained popularity in orthopedic surgeries due to their superior biocompatibility, enhanced corrosion resistance, and lower modulus compared to stainless steels and cobalt-based alloys.

Iron is an indispensable chemical element in the human body and possesses favorable mechanical properties, high biocompatibility, and a slow degradation rate [209]. Its high elastic modulus is associated with high radial strength. However, the degradation rate of Fe is too slow for it to be widely employed in tissue engineering. Further investigations are needed to achieve a desirable corrosion rate, and Fe material properties must be adjusted for it to be suitable for biomedical purposes [210]. Strontium (Sr) is also considered a promising biomaterial with distinct properties that can influence tissue regeneration processes [211], where it was reported that Sr increases osteoblast activity and increases bioactivity when incorporated with HA lattice [212]. Zinc plays a crucial role in various biological functions, including nucleic acid metabolism, DNA synthesis, enzymatic reactions, and apoptosis regulation. It is present in different body parts, such as the skin, liver, bones, and muscles. Mg plays a crucial role in various bodily functions. The Mg ion (Mg^2+^) acts as a cofactor in over 300 enzymatic reactions, including protein and DNA/RNA synthesis, ion transportation, cell migration and function, and intracellular energy production through the ATP system [213,214]. The interaction between an absorbable metal and human body fluid may lead to the initiation of the anodic reaction, which is accompanied by the generation of electrons, which are subsequently consumed by the cathodic reaction. For mg-based alloys, the cathodic reaction involves water reduction, while for Zn-based alloys and Fe-based alloys, it involves the reduction of dissolved oxygen. In a physiological environment, the presence of high chloride ion concentrations results in the breakdown of the degradation layers and accelerates the degradation process. Depending on the size of the degradation particles, macrophages and/or fibrous tissue may encapsulate these particles until complete degradation of the metal occurs [215].

In the next sections, we will delve into the specific properties and applications of magnesium and zinc as biomaterials, building upon their essential roles in various biological functions in soft tissue regeneration and skin expansion.

#### 6.3.1. Magnesium (Mg)

The total Mg^2+^ content in the normal adult body is estimated to be around 25 g, with approximately 53% found in the bone, and the extracellular Mg^2+^ accounts for about 1% of the total Mg^2+^ content [216]. Magnesium plays a vital role in numerous biological processes and is essential for sustaining life. Mg^2+^ is involved in enzymatic reactions through two key interactions: (1) binding to the substrate, forming a complex that interacts with the enzyme, such as Mg ATP in ATP-utilizing enzymes, and (2) binding to the enzyme itself, acting as an allosteric activator [217,218]. Magnesium ions possess a small size that enables them to permeate the skin, resulting in reduced inflammation, enhanced water binding to the skin, and accelerated repair of the skin barrier [219,220]. The improvement of skin barrier function relies on minimizing trans-epidermal water loss, maintaining a hydrated stratum corneum, and minimizing inflammation [221]. Notably, a previous study specifically examined the impact of magnesium salt on skin barrier recovery and concluded that it effectively expedites the healing process of the skin barrier [222,223,224]. Magnesium ions have garnered significant attention in the context of skin substitutes due to their ability to promote skin repair. Among the various forms of magnesium, magnesium oxide, an inorganic metal oxide, stands out as it releases soluble Mg^2+^ ions that play a crucial role in cellular processes [225]. These Mg^2+^ ions actively participate in wound healing by facilitating the recovery of damaged tissue cells, regulating cellular metabolism, and controlling enzyme activity. This mechanism enhances the healing process following traumatic injuries [226]. Additionally, magnesium influences the migration of keratinocytes, regulates epidermal differentiation and proliferation, and exhibits anti-inflammatory properties [227]. It also plays a role in maintaining the barrier function of the skin, thereby controlling the hydration of the stratum corneum [227]. Considering these factors, improving the skin barrier function of compromised skin holds paramount importance for magnesium. On the other hand, Mg deficiency can reduce parathyroid hormone (PTH) secretion, which, in turn, results in lower vitamin D levels [228]. Researchers have found that post-menopausal women who are vitamin D deficient have lower levels of PTH and Mg than those who are not deficient [229].

On the cellular level, magnesium has been shown to stimulate collagen synthesis in cultured fibroblasts, indicating its potential role in promoting connective tissue formation [230]. On the other hand, it also inhibits prolyl and lysyl hydroxylases [231]. Additionally, studies have indicated that Mg^2+^ is specifically associated with the elastin core within elastic fibers rather than with the associated microfibrils known as oxytalan fibers [232,233]. Elastin degradation is a significant process in various physiological events, including growth, wound healing, and tissue remodeling [234]. The association of Mg^2+^ with elastin core suggests that it plays a crucial role in safeguarding the extensibility of elastin, contributing to its mechanical properties. Therefore, it seems that Mg^2+^ is not only involved in maintaining the structure and mechanical integrity of elastic fibers but also actively participates in the elastolysis of these fibers [235]. In cartilage, magnesium-associated proteoglycans play a crucial role in preventing tissue swelling and degradation. According to a previous study [236], it has been observed that decorin proteoglycans, which are found in proximity to collagen fibers, have the ability to interfere with TGFβ/smad dependent transcriptional processes in human mesangial cells [237], where Mg^2+^ has the potential to exert its effects at the protein kinase II level. Furthermore, magnesium regulates the functional activity of integrins [238]. The modulation of integrins, which are involved in cell adhesion to extracellular matrix components, has been found to play a crucial role in facilitating cell migration [239,240]. Studies have reported that the presence of Mg^2+^ can enhance the adhesion of keratinocytes and fibroblasts to type I collagen and laminins (glycoproteins found in the basement membrane). Interestingly, this effect is dependent on the concentration of Mg^2+^, while the presence of Ca^2+^ counteracts this enhancement [241,242]. Cell migration may be facilitated by the interaction between integrins and MMPs if Mg^2+^ is able to modulate the activity of MMPs and cause changes in integrin conformation [243]. These diverse properties associated with magnesium make it serve as a key player in physiological and pathological situations involving connective tissue and matrix-associated cells. Magnesium is a highly performant biodegradable metal that possesses significant properties [244]. When ingested in quantities of 350 mg per day, 25 mg is deposited in the human body, with half of it in bones, while the remainder is excreted in urine [225]. Its density is around 1.7 g/cm^3^, while its Young’s modulus is approximately 42 GPa, making it quite similar to that of human bone (density of 1.95 g/cm^3^ and Young’s modulus ranging between 3 and 20 GPa) [245]. Magnesium biodegradation inside the human body leads to a rapid degradation process, which creates gas pockets and eliminates hydrogen. This results in a local alkalization process near the scaffold structure, leading to an increase in hydroxyl (OH-) ions [246]. The presence of these ions can worsen the physiological microenvironment and even cause alkaline poisoning effects at pH levels above 7.8. Moreover, if the evolution of hydrogen gas is generated in high quantities, it may lead to tissue damage and other complications [247]. The accumulation of hydrogen gas in the tissue has the potential to harm the tissue, cause inflammation, and make the patient uncomfortable. Although the body may safely absorb and tolerate tiny amounts of hydrogen gas, excessive accumulation can have negative effects [248]. According to a previous study, the Mg ions were not toxic to the kidneys or liver, and no major bone healing issues were reported. However, the emitted gas containing H_2_, CO, and CO_2_ can result in problems such as long-term osteolytic lesions and superficial skin necrosis [249]. On the other hand, excessive magnesium intake might cause health concerns. It is crucial to understand that magnesium overdose is rare and usually affects those who have kidney problems or who take supplements with exceptionally high dosages of the element [250]. Too much magnesium can cause gastrointestinal problems such as nausea, vomiting, and diarrhea [250]. These symptoms are usually the body’s way of getting rid of excess magnesium. High doses of magnesium can also lower blood pressure (hypotension), which can cause dizziness, nausea, or even tremors in extreme cases [251]. In severe cases, magnesium toxicity can affect the respiratory system, making it difficult to breathe [250]. Before the widespread application of magnesium as a biomaterial, it is crucial to obtain a comprehensive understanding of its corrosion behavior and point out the link between these health issues and its long-term, excessive use. Although the correlation between in vivo and in vitro corrosion behavior is not yet quantitatively established, corrosion tests are commonly conducted during the initial stage of alloy development to avoid costly in vivo animal trials [252]. Several published papers have concentrated on exploring the impact of individual components present in different media on the corrosion of magnesium [253,254]. Representative test media include SBFs (simulated body fluids), cell culture medium, and protein-containing media [255,256].

The impact of individual inorganic ions found in the simulated body fluids, such as Cl^−^, carbonate, phosphates, sulfate, and Ca^2+^, on the rate of corrosion has been extensively studied. It has been found that the presence of carbonate and phosphates can slow down the corrosion rate of magnesium, while the effect of sulfate is not significant [252]. Cell culture medium, for example, SBFs, is a mild corrosive medium that tends to minimize differences in corrosion rates between various magnesium alloys.

Moreover, proteins have been shown to have various effects on the corrosion of magnesium, either accelerating or inhibiting it. Gu et al. reported that FBS (fetal bovine serum) had the opposite effect on the corrosion of Mg–Ca and AZ series alloys in DMEM [257]. Zhang et al. observed that the corrosion rate of Mg–Nd–Zn–Zr in the M199 cell culture medium was slowed down with the addition of 10% FBS under sterile cell culture conditions [258]. In DMEM, Johnson et al. found that the degradation of pure Mg was minimally affected by the addition of FBS, but the weight loss of Mg–Y increased [259].

Implantation in an animal body leads to alterations in the local physiological environment, such as inflammation, which affects the corrosive environment surrounding the implant. Moreover, mechanical stress during the service period also impacts the corrosion of implanted materials [260,261,262]. These factors cannot be easily represented comprehensively through corrosion tests. Therefore, it is impractical to entirely simulate the in vivo response through corrosion tests [263]. No single corrosion test method can provide complete answers to all the questions regarding Mg corrosion. The corrosion rate measured by such tests cannot fully represent the in vivo corrosion rate, which also varies [264]. Hence, we believe that the main objective of corrosion tests should be to identify the most effective metallic materials, organic and inorganic coatings, as well as various additives such as drugs or corrosion modulators and to understand their interactions. Of course, the criteria for evaluating performance should be based on agreement within the scientific community. Improving the Mg properties involves carefully monitoring and enhancing its degradation rate through surface treatment or alloying with chemical components that lead to a reduction in the amount of hydrogen gas and OH^−^ ions.

##### Magnesium and Its Alloys for Medical Applications

Mg and its alloys have emerged as promising biodegradable metals for medical implants, revolutionizing the use of metal implants in medical settings [265,266]. Currently, magnesium-based implants are widely used in two key applications: vascular interventions [267] and orthopedic procedures [268]. In 2016, BIOTRONIK (Berlin, Germany) introduced the first Mg-based stent that is commercially biodegradable [269]. The biodegradable nature of magnesium and its alloys, which are equivalent to those of polymers, the most widely used materials for such purposes, and their mechanical characteristics, such as density and elastic modulus akin to cortical bone, make them promising.

The degradation of Mg results in changes in the surface chemistry of the implant. This process creates degradation products that dissolve and elevate the pH in the peri-implant region while simultaneously protecting the implant from further degradation [270]. However, for the successful use of biodegradable Mg implants in tissue repair, it is necessary to ensure the proper maintenance of the implant during the healing period. If the degradation of the implant occurs too quickly, it can result in the formation of hydrogen bubbles that interfere with the attachment of proteins and cells to the implant surface, leading to premature implant failure. Therefore, the ideal degradation of Mg implants should start slowly and increase gradually over time once the damaged tissue has sufficiently healed [271].

It was demonstrated previously that the Mg^2+^-incorporated alginate hydrogel showed potential effects on the proliferation and differentiation of osteoblasts [272]. Another study by Roh et al. reported that the addition of MgO and Hap to the 3D PCL scaffold positively influenced various behaviors of pre-osteoblast cells, including initial adhesion, proliferation, and differentiation [273]. Additionally, Yuan et al. demonstrated that the bioresorbable microspheres made up of poly(lactide-co-glycolide) (PLGA) co-embedded with MgO and MgCO_3_ affect the efficiency in treating bone defects [274]. These findings suggest that the controlled delivery of Mg^2+^ ions through an appropriate scaffold could be a promising approach to enhance bone regeneration.

Alloying elements have been added to pure Mg to achieve a moderate and homogeneous degradation behavior. According to previous work, the alloy composition plays a crucial role in implant degradation, and in Guinea pig bones, the device should be present for at least 12 weeks to allow for healing [275]. The use of rare earth elements (REE) in Mg implants has yielded the most favorable response in in-vivo applications [275]. REEs, which consist of 17 elements, are commonly added to Mg to enhance its ductility, degradation resistance, and grain boundary strength. In vitro studies by Feyerabend et al. examined the impact of certain REEs on the viability, apoptosis, and expression of inflammatory cytokines in four types of cells. The findings indicated that lanthanum (La) and cerium (Ce) had the highest cytotoxicity, while gadolinium (Gd) and yttrium (Y) appeared to be conducive to promoting cell growth [276].

Some researchers have investigated the potential use of Mg alloys as open-porous scaffolds for load-bearing applications in tissue engineering. Witte et al. created two porous metallic scaffolds by casting an Mg–Al–Zn alloy and an Mg–Al–Zn–Mn alloy and tested them in vivo. Degradation occurred too rapidly, and the presence of Al and Zn caused inflammatory reactions [277]. A wide range of biodegradable Mg-based alloys with varying zinc contents was reported previously, such as Mg–Zn, Mg–Zn–Mn–Ca, Mg–Zn–Y, and Mg–Zn–Si [278,279,280]. In conclusion, the use of Mg and its alloys as biodegradable metals for medical implants has brought about a significant revolution in the field. Despite the promising properties of magnesium, research on its applications in skin expansion and soft tissue regeneration is still relatively limited. Further studies are needed to explore and understand the full potential of magnesium in these areas. The continued innovation and improvement in the field of magnesium and its alloy implants are paving the way for a wider integration of these devices across many therapeutic applications. These substances not only improve biodegradability but also display better mechanical qualities, opening the door to significant developments in the field of tissue regeneration and repair. As we explore the potential that magnesium-based materials can provide for the advancement of healthcare practices, the future does indeed seem hopeful.

#### 6.3.2. Zinc (Zn)

The essential micronutrient zinc plays a vital role in supporting immune function. Despite the human body containing a total zinc amount of 2 to 4 g, there is no specialized storage system [281]. Consequently, a daily intake of zinc is necessary to maintain a steady state for optimal immune function. Zn obtained from dietary sources is absorbed in the small intestine and distributed through plasma. However, the concentration of zinc in the plasma is relatively low, measuring around 90 µg/dL, which accounts for less than 1% of the body’s total zinc content [282]. Although the plasma zinc pool is small, it holds significant immunological importance. Zinc primarily exists as an intracellular ion, with distribution occurring between various cellular compartments [283]. This distribution includes the cell nucleus (30–40%), cytoplasm, organelles, and vesicles (50%) [284]. Notably, there are specialized zinc-containing vesicles known as “zincosomes” that can store high levels of zinc upon stimulation [285,286].

Similar to plasma zinc, the majority of cellular zinc is tightly bound to proteins, resulting in a small portion of intracellular zinc remaining unbound or loosely bound, known as free zinc. Recent research has identified approximately 4000 proteins and a similar number of transcription factors that possess zinc-binding motifs [287,288]. The zinc tightly bound to proteins plays a crucial role in the catalytic, cocatalytic, and structural functions of enzymes [284]. It contributes to the stabilization of structural domains, such as zinc fingers and related structures, and facilitates protein–protein or protein–nucleic acid interactions, as observed in numerous transcription factors [289].

Throughout evolution, efficient mechanisms for maintaining zinc homeostasis have developed to prevent excessive accumulation of this essential micronutrient. These mechanisms involve two families of eukaryotic zinc transporters known as Zip and ZnT. The Zip family, also referred to as the Zrt-like, Irt-like Protein family, consists of 14 genes designated as solute carrier family 39 (SLC39) A1 to A14. Zips are responsible for transporting zinc into the cytosol. On the other hand, the ZnT family, comprising 10 genes (SLC30A1-10), facilitates the transport of zinc in the opposite direction. These transporter families not only regulate zinc levels within the cytosol but also play a role in controlling zinc distribution within cellular compartments such as the endoplasmic reticulum, mitochondria, and Golgi apparatus [290,291]. Additionally, aside from these transporter-mediated processes, zinc uptake can occur through diffusion involving amino acids, calcium-conducting channels, and various receptors [292,293]. Alterations in the concentration of free zinc within cells can impact signaling pathways, ultimately leading to modifications in cellular responses. This connection between intracellular zinc levels and signaling pathways has been demonstrated in various studies [286,294,295]. Additionally, cellular activation and stimulation can induce fluctuations in intracellular zinc levels [296]. This suggests a potential interaction between zinc homeostasis and signal transduction, implying that zinc may play analogous roles to calcium, which is a well-known second messenger [297].

Zinc is present both intracellularly and in the ECM of epidermal and dermal tissues, where it plays various crucial roles [298]. In human skin, the concentration of zinc is higher in the epidermis (50–70 μg/g dry weight) compared to the dermis (10–15 μg/g dry weight). This difference may reflect the involvement of zinc-dependent RNA and DNA polymerases in the mitotically active basal cells [299,300,301,302]. Immunohistochemical and in situ hybridization localization studies have revealed that in normal skin, high levels of metallothionein (MTs) are found in the basal epidermis, while their concentrations are reduced in postmitotic keratinocytes, reticuloendothelial cells, and fibroblasts [303,304,305]. The presence of MTs is associated with increased tissue zinc concentrations [305], and skin lacking MTs exhibits significantly lower zinc content compared to wild-type mice [303].

The interplay between zinc and calcium is crucial for basal cell mitosis and postmitotic maturation processes in normal skin, which involve keratohyalin synthesis and keratinization [306,307]. In thin hairy skin, where mitosis is inversely related to hair coverage, the levels of zinc and calcium are noticeably lower [308]. In sensory epithelia such as the nasal mucosa and tongue, higher levels of zinc are observed. These elevated zinc levels not only correlate with high mitotic activity, prolonged zones of keratinization, and abundant protein-bound phospholipids but also underscore the importance of zinc in taste and smell perception [309].

The inclusion of zinc ions can accelerate numerous biochemical and molecular processes involved in wound repair by promoting the up-regulation of MTs and zinc metalloenzymes [305,310]. Moreover, zinc overdose is a rare condition that often happens when people use excessive amounts of zinc supplements over an extended period [311]. High zinc dosages may result in nausea, vomiting, and diarrhea. These signs typically appear shortly after consuming too much zinc [311]. Chronic zinc overload may weaken the immune system, increasing the susceptibility to infections [312]. The risk of heart disease may also rise if zinc intake is too high because it lowers levels of “good” high-density lipoprotein (HDL) cholesterol [313]. On the other hand, any deficiency in the expression of zinc-finger transcription factors in the mRNA coding of growth factors is indicative of compromised wound healing [314,315]. The modulation of zinc metabolism through the regulation of MT genes is attributed to the influence of Interleukin-1 (IL-1) [316]. This mechanism provides a potential explanation for the significant rise in zinc levels during the initial inflammatory phase of experimental wounds [304,317,318]. In a rat wound model, it was observed that zinc levels in the wound margin escalated by 15–20% within 24 h and further increased to 30% during the peak formation of granulation tissue and epidermal proliferation [317]. The functional significance of zinc in repair systems is supported by the presence of zinc metalloenzymes such as RNA and DNA polymerases, alkaline phosphatase, and MMPs, indicating their involvement in various biological processes [319]. Additionally, the expression of integrins α2β1, α3β1, α6β4, and αvβ5 is influenced by zinc, thereby regulating keratinization and keratinocyte migration. In normal skin, these integrins are predominantly expressed in the basal layer and play a crucial role in intercellular and cell-basement membrane adhesion [320]. However, their expression is altered in response to inflammation or tissue injury. The addition of supplementary zinc promotes the induction of α2, α3, αv, and α6 integrin subunits, which in turn affect keratinocyte motility during the healing phase [320]. Accordingly, doses and releases are carefully optimized to ensure therapeutic benefit while avoiding any adverse health effects associated with overdose.

Zn shows promise as a material for manufacturing medical implants. The cytotoxicity of zinc has been investigated in previous studies on various human and animal cell types to confirm this biocompatibility [321,322,323,324,325]. Wu et al. [326] reported a 70% viability of human endometrial epithelial cells when exposed to 150 μmol/L Zn. Conversely, rat retinal cells showed a similar reduction in viability at a lower concentration of 50 μmol/l Zn [327]. Human proximal tubular cells exhibited a viability of less than 50% when exposed to a 100 μmol/L Zn solution [328]. In a recent study by Cheng et al. [329], it was found that 1 μg/mL (equivalent to 15 μM) of Zn did not exhibit toxicity to ECV304 cells, but it decreased the viability of L929 cells in the same environment.

Initial experiments have indicated that Zn-based medical implants pose minimal risk of toxic side effects, as their absorption rate remains well below the tolerated threshold of approximately 15 mg/day due to the moderate degradation rate of Zn [321,323,325]. Zn-based implants exhibit sufficient structural longevity in the body, primarily attributed to the formation of a protective layer of Zn oxide (Zn(OH_2_) and ZnO) on the implant surface [330]. This protective layer resists corrosion during the initial 3 months following implantation, while corrosion rates tend to accelerate around 4.5 to 6 months as the layer thickness increases [325]. The degradation behavior of Zn demonstrates favorable mechanical integrity during the early stages of implantation (4–6 months) and suitable absorption periods (1–2 years) after the healing process [325,331]. Moreover, zinc in vivo experiments were conducted by Bowen et al. [325] and Li et al. [332], demonstrating significant advancements in this area. Bowen et al. conducted a study where they implanted thin zinc wires into the abdominal aorta of adult rats to examine corrosion rates, corrosion products, and tissue adherence. The results revealed a corrosion rate ranging from 10 to 50 μm/year, which exhibited a progressive increase over a six-month period. Additionally, it was noteworthy that the implant remained structurally intact for a minimum of four months following implantation [325,332].

Beyond its role in nutrition and biocompatibility, zinc has drawn interest in the field of biomaterials because of its natural antibacterial characteristics [333]. There are numerous mechanisms through which zinc exerts its antimicrobial effects. One important process includes rupturing bacterial cell membranes, which can cause cell death and the release of cellular contents. Furthermore, zinc ions can enter bacterial cells and disrupt vital biological functions, including protein synthesis and DNA replication [334,335]. Zinc is effective against a broad spectrum of bacteria, including both Gram-positive and Gram-negative strains [336]. The antibacterial properties of zinc not only defend against infections but also help biomaterial biocompatibility [337]. Improved patient outcomes and lower medical expenses can result from decreased infection rates and related consequences.

##### Zinc and Its Alloys for Medical Applications

Zinc and its alloys possess specific properties and degradation characteristics that offer potential solutions to the challenges limiting the broader use of magnesium (Mg) and iron (Fe)-based alloys for biodegradable implants. The corrosion rate of pure Zn falls within the range between pure Mg and Fe while also avoiding the release of hydrogen gas during in-vivo degradation processes [268]. Zn-based implants are demonstrated to sustain their mechanical integrity for the initial six months post-implantation. Furthermore, stress accelerates localized corrosion, leading to faster degradation during the recovery phase [338]. Furthermore, the degradation products that arise from the corrosion of zinc exhibit a compact nature and are biocompatible. Additionally, Zn possesses a comparably low melting point of approximately 419.75 °C and exhibits low chemical reactivity. These characteristics simplify the manufacturing processes involved in producing Zn-based products, including casting and thermo-mechanical processing, in comparison to other biodegradable and conventional permanent implant metals [339]. Nonetheless, the widespread use of Zn-based biodegradable materials is hindered by their inadequate mechanical properties, which encompass insufficient mechanical strength and ductility [324,340,341]. Previous studies have explored the implementation of thermo-mechanical processing techniques to enhance the mechanical properties of Zn [323,340,341]. Pure zinc processed at room temperature (RT) has been found to fall short of meeting the necessary mechanical criteria for load-bearing implants [342]. To enhance the mechanical properties of zinc alloys, different alloying elements have been investigated, including Mg, aluminum (Al), Ti, copper (Cu), calcium (Ca), silver (Ag), strontium (Sr), and manganese (Mn). These alloying elements aim to improve the mechanical properties of Zn alloys through mechanisms such as grain boundary strengthening, solid solution strengthening, dispersion strengthening, and precipitation strengthening [324,342,343].

Copper is a favorable choice for alloying with zinc due to its relatively high solubility in Zn at the melting temperature, reaching up to 2 wt%. The improved mechanical properties of Zn–Cu alloys stem from the effects of solid solution strengthening and grain boundary strengthening exerted by Cu [344]. When the Cu content in Zn–Cu alloys is below 1%, they exhibit a nearly single-phase microstructure. As the Cu content increases, the ductility of Zn–Cu alloys is further enhanced, although the increase in mechanical strength becomes relatively minor [345]. Extensive research has been conducted to investigate the impact of Mg additions on the mechanical properties of Zn alloys [346]. The mechanical improvements observed in Zn–Mg alloys are primarily attributed to the presence of brittle phases, namely Mg_2_Zn_11_ and MgZn_2_ eutectic phases, as the solubility of Mg in Zn is low (0.1 wt% at temperatures above 300 ℃ and nearly zero at RT). Notably, Zn alloys containing small amounts of Mg (<1%) exhibit an exceptional balance of strength and plasticity compared to other investigated Zn alloys [325]. However, the conventional casting and processing methods used for Zn alloyed with low Mg content (<1%) are unable to achieve the mechanical properties required for load-bearing biodegradable implants [347,348]. Hence, the processing of Zn–Mg alloys at room temperature presents a promising approach for manufacturing Zn–Mg products with enhanced mechanical properties.

#### 6.3.3. Zn–Mg Alloys

Zn alloyed with Mg has emerged as a promising material for biomedical applications. It is crucial to adjust the ratio between these ions to achieve targeted tissue responses and improve the healing process. There are various methods for adjusting the Mg and Zn ion ratios in biomaterials for various clinical uses [333,349]. Ion release rates must be carefully controlled in order to produce the intended therapeutic benefits while minimizing potential cytotoxicity or unfavorable tissue reactions [350]. Mg and Zn ion release kinetics can be effectively altered by adjusting various factors, including material composition, surface coatings, and fabrication processes [351]. For instance, a sustained release of both ions over time can be achieved by developing Mg-rich alloys with precisely controlled Zn content. Moreover, depending on the targeted tissue and its regenerating needs, the ideal Mg–Zn ratio can significantly change. A higher Mg content in the implant may be preferred in the field of bone tissue engineering due to the well-known function of Mg in bone growth and mineralization [352]. Zn-rich materials, on the other hand, can be preferred for soft tissue applications where wound-healing and antibacterial capabilities of Zn are useful [201,353]. Moreover, the optimal Mg–Zn ratio can also vary from patient to patient, depending on their age, general health, and underlying illnesses. This personalized method can have the promise of promoting improved tissue healing and clinical outcomes across a wide range of medical applications.

According to Yao et al. [354], Zn alloys containing Mg compositions below 10% exhibit significantly enhanced corrosion properties compared to pure Zn. The enhanced corrosion resistance of the implanted Zn–Mg alloy enables it to retain its mechanical integrity for a sufficient period, facilitating tissue repair. The favorable corrosion resistance is primarily attributed to the presence of intermetallic phases, namely Mg_2_Zn_11_ and MgZn_2_, which facilitate the formation of an electrochemically inert protective film, such as Mg_2_(OH)_2_CO_3_, on the surface of the Zn–Mg alloys [343]. Notably, as-cast Zn–3 wt%Mg alloys have been reported to exhibit a refined nanostructure and favorable corrosion resistance [279]. Additionally, studies have indicated that Zn alloyed with 1 wt% Mg achieves an optimal balance between strength and ductility [355]. Previous research has suggested that higher Mg contents (≤3 wt%) result in increased volume fractions of the eutectic phase, leading to improved strength and hardness but reduced ductility [343,356]. The limited ductility observed in Zn–Mg alloys is considered inadequate for medical implant applications, and the non-uniform breakdown due to preferential corrosion of Mg has hindered the widespread use of these alloys in biomedical applications [357,358]. Therefore, increasing research attention has been focused on Zn alloys containing low amounts of Mg (≤1 wt% Mg) in order to reduce the presence of intermetallic phases while incorporating well-designed processing techniques to enhance mechanical and corrosion properties [323]. Gong et al. [356] conducted a study showing that hot extrusion enhances the uniformity of biodegradation and mechanical properties in Zn–1 wt% Mg alloys. In vitro cytotoxicity tests also confirmed the excellent biocompatibility of the alloy. Additionally, several studies have investigated the mechanical properties, corrosion properties, and cytotoxicity of Zn–1 wt% Mg alloys alone or in combination with other elements such as Mn, Ca, and Sr, employing various fabrication techniques [343,356,359].

Li et al. [332] demonstrated the high viability of ECV304, VSCM, and MG63 cells when exposed to extracts from extruded Zn–1 Mg alloy. Additionally, they found that ECV304 and MG63 cells exhibited healthy behavior when directly cultured on the surface of Zn–1 Mg alloy for 24 h. In contrast, VSCM cells displayed an unhealthy and deceased morphology after 24 h on the surface of Zn–1 Mg alloys [332]. Furthermore, Gong et al. observed excellent viability of L-929 cells in diluted extracts (1:15) prepared with DMEM and 10% FBS after 24 and 72 h [356]. Initial in vitro cytotoxicity assessments were conducted on as-cast Zn alloys, and the results demonstrated that U-2 OS cells exposed to the extract from Zn–0.8 Mg alloys containing 70 μmol/L Zn exhibited a viability of 80% [360]. Murni et al. [361] conducted a study on the cytotoxicity of Zn–3 Mg alloy. Extracts were prepared by incubating 0.75 mg of Mg–3Zn powder in cell culture for 72 h, and the resulting extract from Zn–3 Mg contained 0.49 ppm of Zn ions. Li et al. [332] conducted a study affirming that the utilization of Zn–1 Mg alloys in mouse femora does not adversely impact the well-being of the mice. In fact, the study observed a strong development of new bone throughout the process [362].

In another study, the implantation of Mg and Zn ions was utilized to enhance the soft tissue sealing ability of Ti [363]. The introduction of these ions resulted in changes in the surface wettability of titanium, impacting the adsorption of proteins on the sample surfaces. Characterization of the physicochemical and biological properties of the ion-implanted samples revealed that both Mg and Zn ions facilitated the accumulation of ECM components such as collagen-I and fibronectin and improved cell adhesion, migration, and proliferation of HGFs [363]. Specifically, the release of Mg^2+^ from Mg ion-implanted samples promoted improved adhesion and motility of human gingival fibroblasts (HGFs), potentially through the regulation of ITGB1 expression and activation of the MAPK signaling pathway [363]. On the other hand, the release of Zn^2+^ resulting from Zn ion implantation primarily enhanced the proliferation of HGFs, which could be attributed to the upregulation of ZIP7 and ZIP13 expression and activation of the TGF-β signaling pathway [363].

## 7. Experimental Studies of Magnesium and Zinc in Soft Tissue Engineering

Magnesium and zinc exhibit distinctive characteristics that make them highly appealing for biomedical applications, including soft tissue engineering and skin expansion. The role of zinc in wound healing has been unequivocally demonstrated in several studies [201,364]. Topical zinc therapy has been shown to effectively reduce wound debris and promote epithelialization in surgical wounds in rat models [365]. Observations of reduced wound debris and necrotic material following topical zinc application in wounds of various origins have led researchers to investigate the action of zinc-dependent MMPs in cultured necrotic tissue from porcine wounds [366]. In vitro experiments using zinc oxide have shown that it enhances the enzymatic breakdown of collagen fragments through the activity of MMPs, which exhibit substrate specificity for various ECM molecules [367,368]. Additionally, locally applied zinc oxide has been found to enhance the repair of ulcerated skin [369]. On the other hand, blocking MMPs has been demonstrated to considerably prolong the wound-healing process [370]. These findings highlight the role of zinc-dependent MMPs in promoting the breakdown of collagen fragments and the repair of damaged skin [371]. In another study, the administration of zinc via intraperitoneal injection immediately after surgery and daily for 4 days (at a dose of 2 mg/kg/day) was found to increase the bursting pressure of colon anastomoses on the seventh day after surgery in both normal rabbits and rabbits treated with a chemotherapeutic agent. Furthermore, the zinc-treated rabbits exhibited increased infiltration of fibroblasts and enhanced epithelialization [298]. However, when these beneficial effects were applied using intraperitoneal zinc sulfate on colon anastomosis repair in a rat model, different results were observed either on the third or seventh day after surgery [372]. In contrast, the research on the role of magnesium in soft tissue engineering and wound healing has been limited. However, new targeted interventions can be investigated by gaining a deeper understanding of the mechanisms underlying the effects of both zinc and magnesium on wound healing and soft tissue regeneration. These interventions have the potential to harness the benefits of these metals, facilitating the healing process and enhancing clinical outcomes.

## 8. Conclusions

Medical implants are surgically inserted into the body to enhance human life by either preserving or restoring functionality in damaged tissues. Nevertheless, the long-term presence of foreign implant materials in the body can lead to persistent detrimental and inflammatory reactions, necessitating secondary surgical procedures for their removal. This introduces additional risks to patients and significantly increases costs. In order to address the limitations of permanent implants, there has been significant research focused on biodegradable implants that are designed to gradually degrade within the body. These biodegradable implants must exhibit excellent biocompatibility, meaning they should not trigger inflammatory responses and must be non-toxic. Furthermore, the implant must effectively fulfill its intended function throughout the entire recovery period. Nowadays, the majority of implants are permanent and comprised of metallic components. Therefore, the base metals, alloy elements, and corrosion products formed during degradation must be non-toxic.

In the realm of soft tissue engineering and skin expansion, two elements, magnesium, and zinc, have emerged as particularly promising candidates. These two elements exhibit distinctive characteristics that render them appealing for biomedical applications. However, despite their potential, they have not been extensively explored in these specific areas of research.

Magnesium exhibits excellent biocompatibility and biodegradability, making it an ideal candidate for medical implants. The biodegradable characteristic of Mg enables gradual decomposition, eliminating the necessity for surgical removal of the implant. Magnesium alloys have shown promising results in orthopedic procedures and vascular interventions. They possess mechanical properties similar to cortical bone, ensuring adequate support and stability during the healing process. Furthermore, magnesium has been found to stimulate collagen synthesis, inhibit fibrotic processes, maintain the extensibility of elastin, and regulate integrin activity. These properties indicate its potential role in promoting tissue regeneration and wound healing.

Similarly, zinc plays a crucial role in various biological processes and is essential for cellular functions such as proliferation, migration, and maturation. It acts as a stabilizer of cell membranes and serves as a cofactor for numerous enzymes. Zinc also influences the expression of zinc-finger transcription factors involved in the coding of growth factors essential for wound healing. Moreover, zinc is involved in the modulation of integrin expression, which affects keratinocyte migration and intercellular adhesion. These attributes highlight the potential of zinc in promoting cell behaviors relevant to tissue engineering and skin expansion.

Despite these promising properties, the specific application of magnesium and zinc in soft tissue engineering and skin expansion has not received extensive research attention. Comprehensive studies examining their effects on cell behavior, tissue regeneration, and the development of suitable scaffolds for controlled delivery are necessary. By addressing these research gaps, magnesium and zinc can be further harnessed as valuable resources for advancing the field of soft tissue engineering and achieving successful skin expansion. Several intriguing directions for future study and development emerge. This includes the potential to improve implant performance, and biocompatibility lies in the ongoing improvement of implant designs, including surface changes and composite materials. It will also be critical to fine-tune the degradation kinetics of magnesium and zinc implants. Additionally, investigating the incorporation of therapeutic substances, such as growth factors or antimicrobial coatings, into implant materials can result in multifunctional implants that can respond to certain clinical needs, such as infection prevention or accelerated wound healing. The field of biodegradable magnesium and zinc implants will advance with the inclusion of these perspectives in future research, ultimately helping patients through improved tissue regeneration, decreased problems, and improved overall quality of care.

In conclusion, this review on the use of magnesium and zinc in skin expansion and soft tissue engineering shows great potential. These substances display a variety of beneficial properties, including biocompatibility, biodegradability, and cellular stimulation. To fully realize their potential in these particular fields, additional comprehensive and in-depth studies are still needed. These initiatives have the potential to transform the field of soft tissue engineering and offer safer and better ways to expand the skin and regenerate tissue.

## Data Availability

No new data were created or analyzed in this study. Data sharing is not applicable to this article.
